# Application of the transient matrix effect for determination of anabolic–androgenic steroids in biological samples by GC–MS/MS

**DOI:** 10.1007/s11419-025-00731-6

**Published:** 2025-06-26

**Authors:** Michal P. Dybowski, Krystian Siwek

**Affiliations:** Department of Chromatography, Institute of Chemical Sciences, Faculty of Chemistry, Maria Curie Sklodowska University in Lublin, 20-031 Lublin, Poland

**Keywords:** Anabolic–androgenic steroids, Transient matrix effect, Blood plasma analysis, Anti-doping, QuEChERS, GC–MS/MS

## Abstract

**Purpose:**

Anabolic–androgenic steroids (AAS) enhance athletic performance, giving athletes an unfair advantage and disrupting fair competition. Banned in sports and listed by World Anti-Doping Agency, they require precise detection. This study aimed to develop a method using the transient matrix effect to improve AAS identification in biological samples.

**Methods:**

Gas chromatography–tandem mass spectrometry (GC–MS/MS) method for determination of AAS samples was developed and validated. Biological samples were prepared using the QuEChERS technique.

**Results:**

The optimised and validated method enhances AAS signals using high-boiling protectants. It ensures good linearity, low detection limits, and reliable precision. Optimal QuEChERS extraction and multiple reaction monitoring transitions in GC–MS/MS were evaluated, confirming applicability with blood plasma samples. The addition of a protectant to the analysed sample results in several notable effects. High-boiling protectants, such as polyethylene glycol (PEG-400), tetradecanoic acid (C14-COOH), *n*-tetradecylalcohol (C14-OH), and *n*-tetradecylamine (C14-NH₂), significantly enhance AAS’s signal in blood plasma. This enhancement, however, is accompanied by a transient matrix effect induced by the protectants. PEG-400 produced the most substantial signal increase, with the response for nandrolone rising by as much as 912%.

**Conclusions:**

The results demonstrate the potential offered by the utilisation of PEG-400 as a protectant to generate a transient matrix effect. The outcome of this process is an increased analytical signal from AAS in blood plasma, enabling their identification even at trace concentrations. The methodology developed and applied during the study can be used to reduce the detection limit of steroids and thus improve antidoping measures in sport.

**Graphical Abstract:**

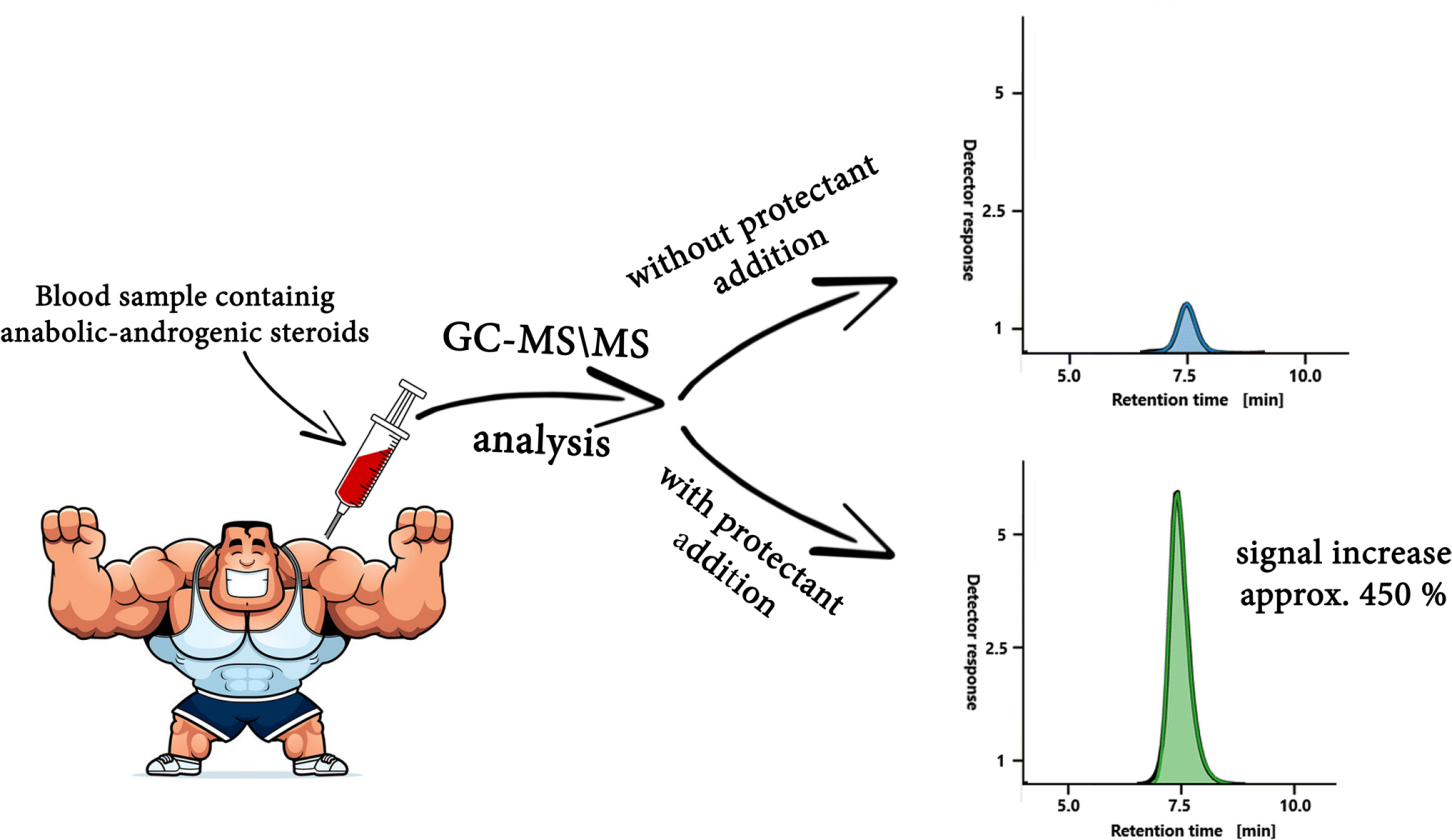

**Supplementary Information:**

The online version contains supplementary material available at 10.1007/s11419-025-00731-6.

## Introduction

According to The Britannica Dictionary, doping is illegal use of a drugs (such as a steroids) to improve an athlete's performance [1]. This definition is likely to be the first association most people have when they hear this term. This is not an unreasonable assumption, as the presence of such substances in the body of athletes is most widely commented in the media. Scandals involving individuals who have been caught using various performance-enhancing substances have attracted the most attention. The use of these substances is incompatible with the principle of fair competition, which is fundamental to the sporting ethos. However, the use of doping, particularly anabolic–androgenic steroids (AAS), has increased in recent years among amateur bodybuilders seeking to accelerate muscle mass gains and this phenomenon is increasingly gaining social acceptance [2]. In order to effectively determine the substances used by the individuals, analytical units specialising in the determination of AAS in biological samples have to continuously improve existing methods, like immunoassay tests or gas chromatography–mass spectrometry (GC–MS), in terms of lowering the limit of detection (LOD) [3]. One potential approach is to reduce or employ the matrix effect for this purpose [4, 5].

The matrix effect, which can be defined as impossibility of quantifying an analyte in a sample due to the presence of other substances or the sample's chemical/physical properties as it is routinely performed, can significantly impact analytical sensitivity and accuracy by GC–MS in complex matrices. The analyte signal may be either increased or decreased as a result of this process [6]. The precise mechanisms of matrix effect still remain unclear, although several theories have been proposed to explain it. These include the possibility that matrix components compete with the analyte to gain charge, blocking it from gaining access to the charge, interfere with its ability to remain charged in the gas phase, or increase the surface tension of the droplet/ the electric resistance [7]. The utilisation of the matrix effect has been largely overlooked in the scientific literature, with the prevailing view being the elimination of this phenomenon [8–11].

However, the matrix effect does not necessarily result in a reduced analytical signal or interfere with the analysis of the substance itself. Instead, it can be employed to enhance the analyte signal by adding a so-called protectant to the system. Our recent study has presented such a procedure [4, 5]. This phenomenon has been termed “transient matrix effect”. This effect was successfully employed to enhance the signal from cannabidiol (CBD) and other xenobiotics in blood plasma (plasma) samples (oleamide was used as a protectant) [4]. The question that arises from this study is whether the matrix effect can also be used to improve the signal from AAS found in plasma samples. If this were possible, would the effect also have a transient nature, and would it significantly alter the analyte signal?

The present work introduces the utilisation of high-boiling protectants to generate a transient matrix effect, with the intention of enhancing the analytical signal of the ten most commonly used AASs (dehydroepiandrosterone, drostanolone propionate, fluoxymestrone, methandienone, nandrolone, testosterone acetate, trenbolone acetate, testosterone, trenbolone and turinabol), determined by gas chromatography–tandem mass spectrometry (GC–MS/MS) in plasma samples.

## Materials and methods

### Materials

Acetonitrile (ACN, HPLC grade), anhydrous magnesium sulfate, sodium chloride were obtained from Avantor (Gliwice, Poland). The Sepra C18-E sorbent (50 µm, 65 Å) used in the QuEChERS procedure was acquired from Phenomenex (Torrance, CA, USA). Standards of AAS (dehydroepiandrosterone, fluoxymestrone, methandienone, nandrolone, testosterone acetate, trenbolone acetate, testosterone, trenbolone) were purchased from Merck (Darmstadt, Germany), with the exception of drostanolone propionate and turinabol, which were obtained from LGC Standards (Teddington, Middlesex, UK). Refrence materials of polyethylene glycol (PEG-400), tetradecanoic acid (C14-COOH), *n-*tetradecylalcohol (C14-OH) or *n-*tetradecylamine (C14-NH_2_) were also acquired from Merck (Darmstadt, Germany). The collection of plasma samples for the purpose of testing was done by a qualified person. All samples were obtained from volunteers. Plasma samples (2 × 5 mL) were collected using a single closed system containing a coagulation activator (according to the manufacturer's instructions, Sarstedt Monovette, Nümbrecht, Germany) and homogenised by mixing them thoroughly. The biological material was then stored in sealed sterile containers at −20 °C (± 2 °C) prior to performing the QuEChERS procedure.

### Standard solutions

The objective of this experiment is to show the impact of high-boiling protectant addition to increase signal from AAS. In order to illustrate it, stock solutions were appropriately diluted with ACN to prepare working solutions:

- AAS solution no. 1 (concentration of dromostanolone propionate, methandienone and testosterone equal 0.1 mg/mL each, concentration of nandrolone, and turinabol equal 1 mg/mL each);
- AAS solution no. 2 (concentration of dehydroepiandrosterone and testosterone acetate equal 0.1 mg/mL each, concentration of fluoxymestrone, trenbolone acetate and trenbolone equal 1 mg/mL each);
- High-boiling protectants solutions (concentration of polyethylene glycol (PEG-400), tetradecanoic acid (C14-COOH), *n-*tetradecylalcohol (C14-OH) or *n-*tetradecylamine (C14-NH_2_) equal 10 mg/mL each).

### QuEChERS sample preparation method (optimal conditions)

An 875 µL plasma sample was transferred to a 2 mL Eppendorf tube and 25 µL of the individual AAS solution (no. 1 or no. 2) was added. The mixture was then subjected to a 30 s vortexing process, after which MgSO₄ (350 mg) and NaCl (87.5 mg) were introduced. After 1 min vortexing period, a cold solution of PEG-400 (100 µL of 10 mg/mL) further diluted in ACN to a volume of 500 μL was introduced to the Eppendorf tube. The entire suspension was immediately vortexed again for 30 s and then subjected to centrifugation at 14,700 rpm for 1.5 min. The resulting supernatant was transferred to a new Eppendorf-type vessel and C18 sorbent (17.5 mg) was added. The sample was then subjected to centrifugation under identical conditions as previously outlined. The aliquot obtained was then analysed.

### GC–MS/MS measurements

Quantitative analyses of analytes were conducted using the GC–MS/MS TQ8040 (Shimadzu, Kyoto, Japan) equipped with a ZB5-Msi fused-silica capillary column (30.0 m × 0.25 mm i.d., 0.25 µm film thickness; Phenomenex). Helium (grade 5.0) was used as the carrier gas, while argon (grade 5.0) was employed as the collision gas. The column flow rate was set at 1.0 mL/min, and 1.0 µL of the sample was injected by an AOC-20i + s type autosampler (Shimadzu). The injector operated in the high-pressure mode (250.0 kPa for 1.5 min; column flow at the initial temperature of 4.90 mL/min) at a temperature of 300 °C. The temperature program applied was as follows: 60 °C for 2 min, followed by a linear increase to 300 °C at a rate of 8 °C/min, and maintenance at 300 °C for 15 min.

### MRM transition for MS/MS detector

The mass spectrometer was operated in Q3-Scan (TIC) mode for the purpose of selecting three characteristic ions for each substances. The mass range that was the target of monitoring was from *m*/*z* 45 to 400 in EI mode (with an ionisation energy of 70 eV and an ion source temperature of 220 °C). GC–MS/MS quantitation for all examined steroids was performed in the multiple reaction monitoring (MRM) mode. Because of that, fragmentation pathways were established for each of them and the energies for the MRM transitions (Table 1).

**Table 1:** MRM transitions and collision voltages of dehydroepiandrosterone, drostanolone propionate, fluoxymestrone, methandienone, nandrolone, testosterone acetate, trenbolone acetate, testosterone, trenbolone and turinabol for gas chromatography–tandem mass spectrometry (GC–MS/MS)

AAS solution type	Compound	Retention time [min]	Qualitative MRM transition [mass > product mass]	Quantitative MRM transition [mass > product mass]	Optimal collision voltage [eV]
AAS solution no. 1	Drostanolone propionate	32.242	360 > 286 360 > 271 286 > 271	286 > 271	6 15 9
	Methandienone	31.223	161 > 147 161 > 134 147 > 134	161 > 134	12 12 12
	Nandrolone	30.038	274 > 256 274 > 231 274 > 207	274 > 256	6 9 9
	Testosterone	30.703	288 > 246 288 > 147 288 > 124	288 > 124	9 18 12
	Turinabol	33.642	334 > 281 334 > 265 334 > 161	334 > 281	6 9 12
AAS solution no. 2	Dehydroepiandrosterone	29.362	288 > 270 288 > 203 270 > 255	288 > 203	6 9 9
	Fluoxymestrone	33.410	336 > 318 336 > 298 278 > 225	336 > 298	18 9 18
	Testosterone acetate	31.727	330 > 288 330 > 228 330 > 124	330 > 124	9 15 15
	Trenbolone acetate	32.055	312 > 270 312 > 252 252 > 237	312 > 252	12 15 15
	Trenbolone	31.097	270 > 252 270 > 226 226 > 198	270 > 226	12 12 12

### Method validation and statistical analysis

The method underwent validation with respect to linearity, the limit of detection (LOD), the limit of quantification (LOQ), and both intraday and interday precision and accuracy. To assess linearity, five replicate analyses were performed at each concentration level tested. The peak areas obtained were subsequently used to generate calibration curves for quantifying all analytes. The LOD and LOQ were determined by spiking plasma with analytical reference standards, followed by injection into the instrument. LOD and LOQ were defined based on the signal-to-noise ratios of 3 and 10, respectively.

Intra- and interday precision, as well as accuracy, were assessed through statistical analysis of the quantitative results obtained on the same day and across three separate days for five independent samples containing the target compounds.

The linearity of the assay was determined using the least squares method and expressed as the coefficient of determination (*R*^2^). Calibration curves were constructed by spiking blank plasma samples with the target analytes, either alone or with the addition of a protectant. The concentration levels of target analytes were as follows:

- 0.1, 1, 5, 10, 25, 50 and 150 ng/mL for testosterone, fluoxymestrone, turinabol, methandienone, trenbolone acetate, dehydroepiandrosterone, trenbolone and dromostanolone propionate;
- 1, 5, 10, 25, 50 and 150 ng/mL for testosterone acetate and nandrolone.

The solutions were prepared in triplicate.

## Results

Before attempting the determination of AAS in real plasma samples, it was necessary to optimise the GC–MS/MS measurement conditions [12] for the determination of these substances. For this purpose, an experiment was carried out to identify three characteristic MRM transitions for each of them and to determine the optimal collision energies for initiating these transitions.

**Fig. 1:**
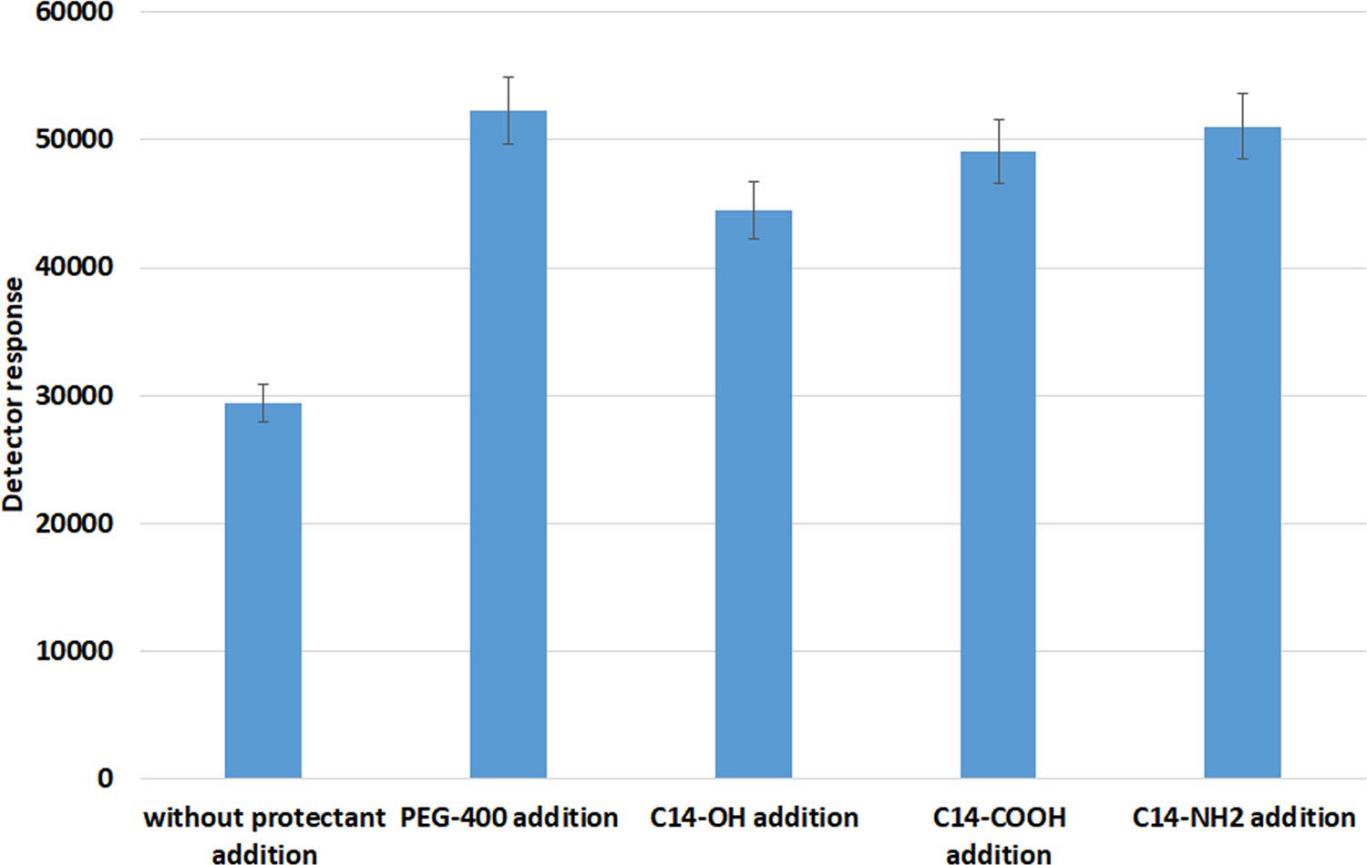
The increment of GC–MS/MS signal magnitude for methandienon after protectant addition

**Fig. 2:**
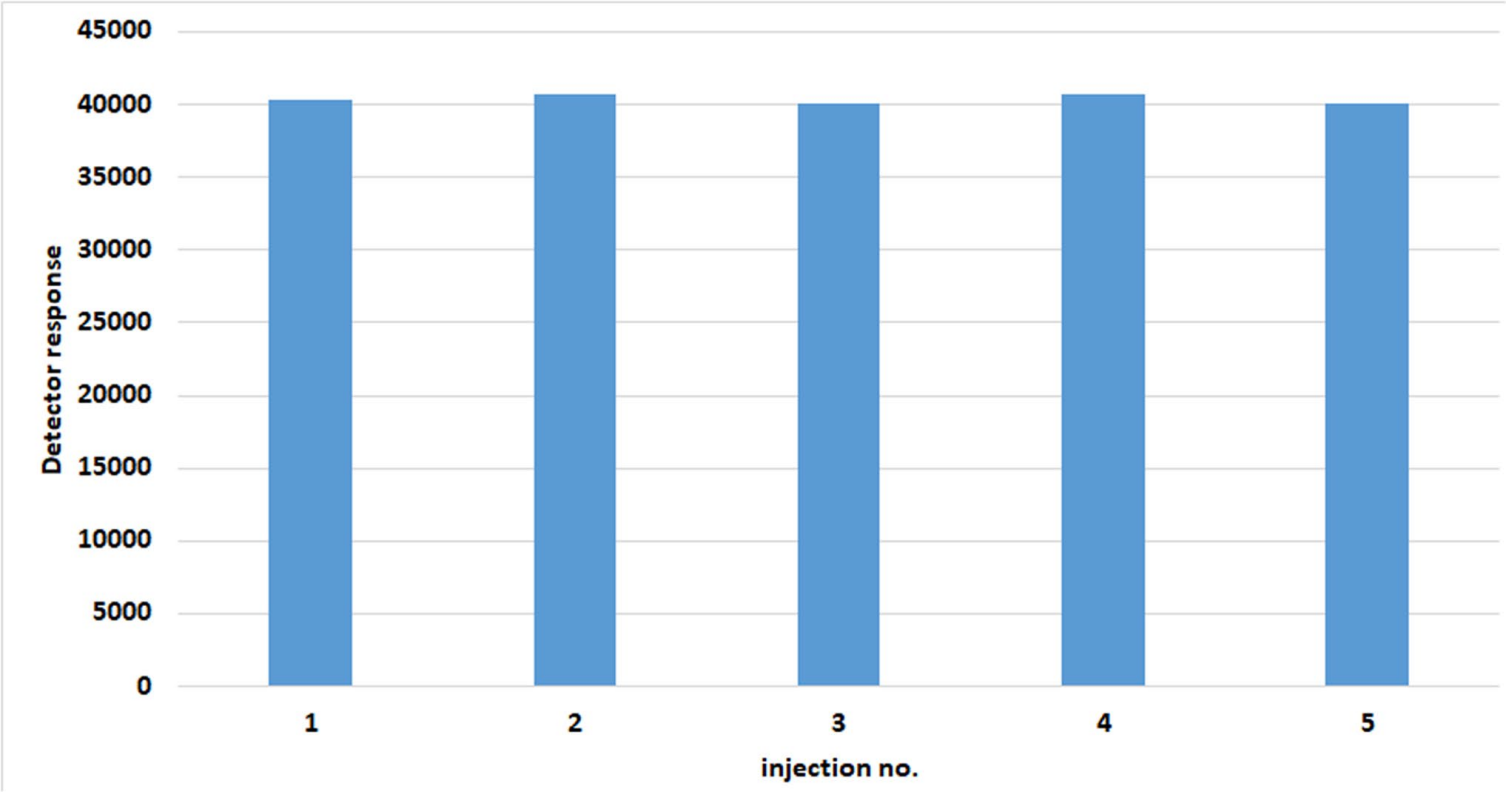
Signal magnitudes from GC–MS/MS data obtained in five consecutive injections of methandienon solution containing PEG-400 as a protectant

**Fig. 3:**
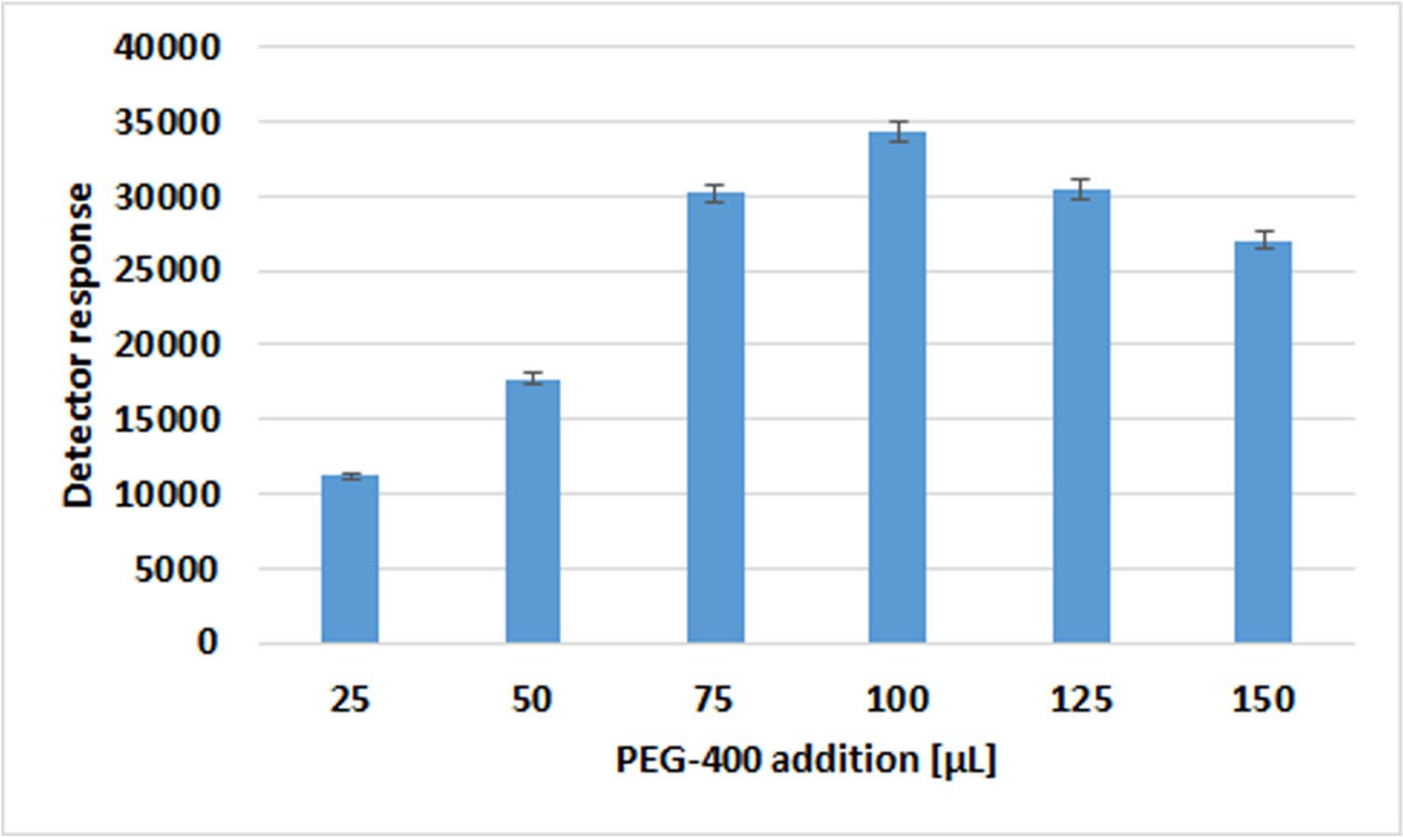
The optimisation of PEG-400 volume addition to the sample in relation to analyte signal magnitude—an example concerning methandienone as a model for AAS analysis

Figure 1S (see supplementary materials) shows the chemical structures and fragmentation pathways of AASs (testosterone, nandrolone and methandienone) selected due to the fact that numerous other steroids are derived from them. The optimal conditions for our instrumentation were optimised for each compound in our laboratory. The MRM transitions and optimal collision energies of the examined compounds are collected in Figs. 2S, 3S (see supplementary materials) and Table 1.

**Fig. 4:**
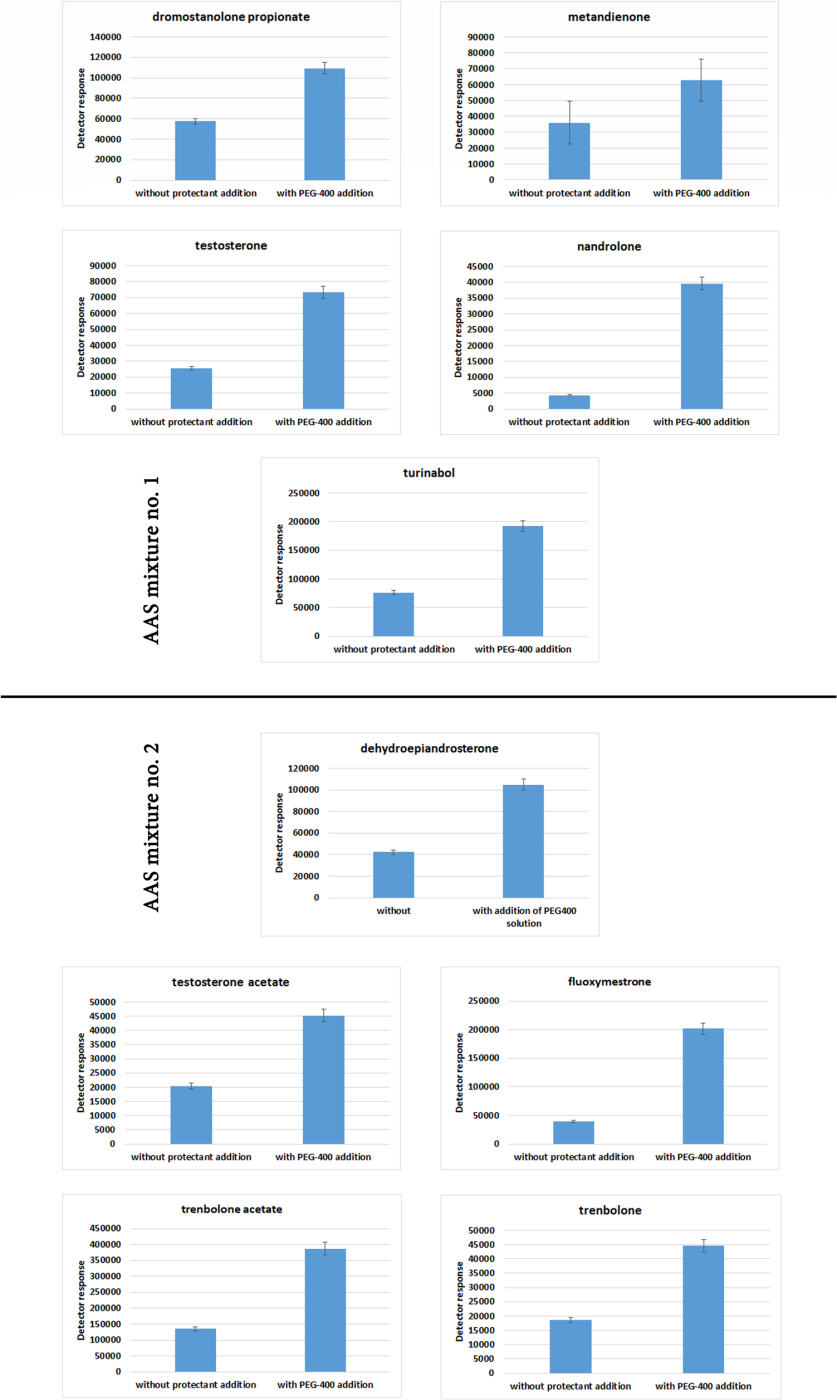
The influence of PEG-400 addition to the sample on examined AAS’s signal magnitude

Presently, the QuEChERS technique is becoming one of the most popular techniques for preparing plasma and whole blood samples for analysis of the substances contained therein [13, 14]. This technique is comprised of two stages: the first involves the extraction of the analytes from the sample matrix and the second - the purification of the analytes, which allows to obtain satisfactory assay results even at trace levels. However, the individual parameters of the method: extraction salts, extractant and sorbent amounts, need to be optimised to achieve the greatest efficiency of the method. The subsequent optimisation stages of the sample preparation procedure are illustrated in Fig. 4S (see supplementary materials), which correspond to the amount of extraction salts (MgSO_4_ and NaCl), the amount of extractant (ACN), the sample volume and the amount of sorbent (C18), respectively. Due to the large number of test results obtained, Fig. 4S includes only those obtained for methandienone.

**Fig. 5:**
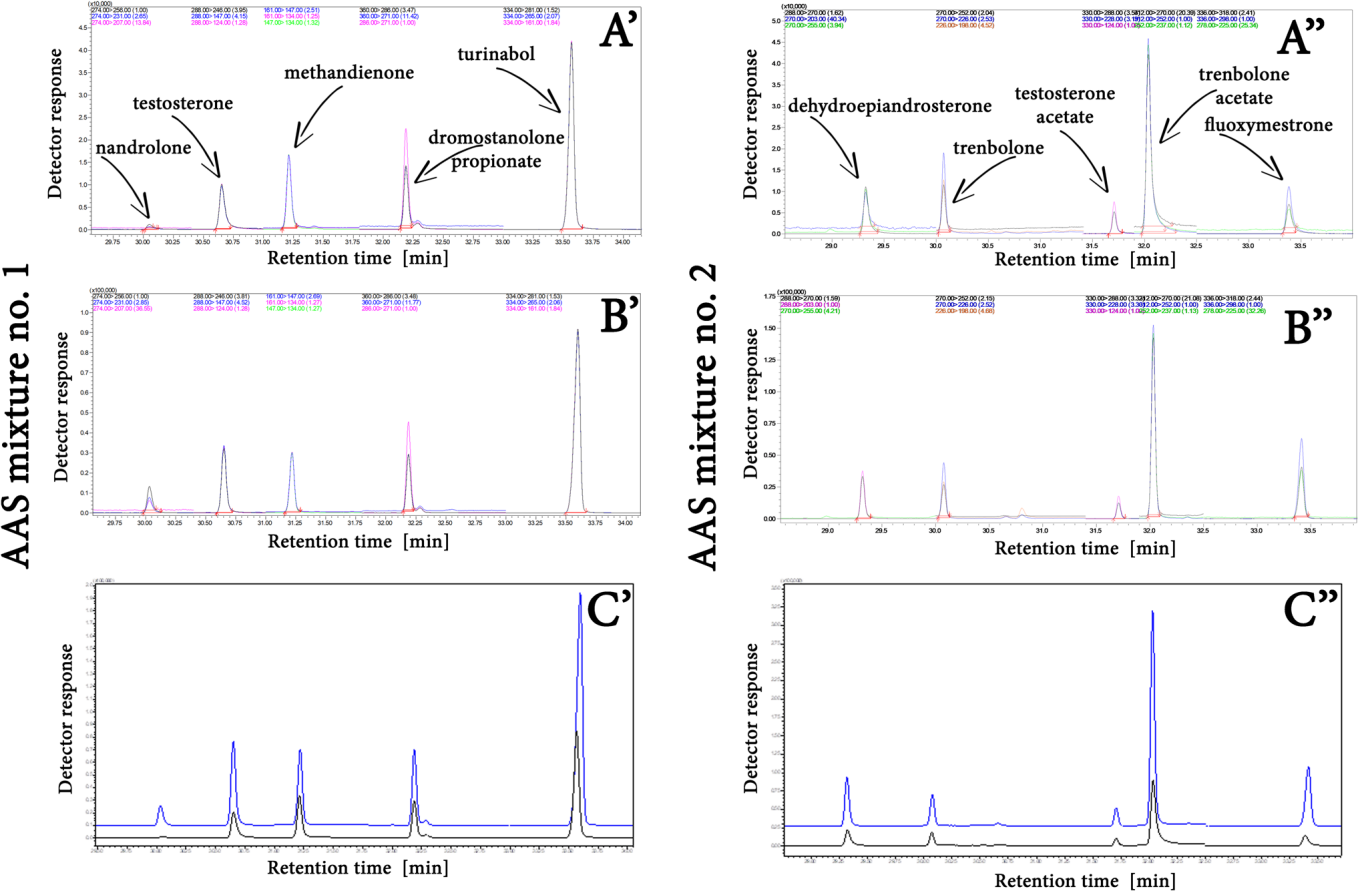
The effect of adding the protectant (PEG-400) to an extract of plasma containing the examined AASs, prepared using the QuEChERS method, prior to GC–MS/MS analysis. (A’ and A’’) -  before protectant addition, (B’ and B’’) -  after protectant addition, (C’ and C’’) -  comparison of chromatograms before (lower) and after (upper) protectant addition

The MRM chromatograms of a blank plasma samples spiked with AAS solutions no. 1 and no. 2 in ACN (see “Standard solutions” section) containing analytes of a concentration of 2 µg/mL (dromostanolone propionate, methandienone, testosterone for solution no. 1 and dehydroepiandrosterone, testosterone acetate for solution no. 2) each, 20 µg/mL (nandrolone, turinabol for solution no. 1 and fluoxymestrone, trenbolone acetate, trenbolone for solution no. 2) each after QuEChERS sample preparation procedure are presented in Fig. 5S (see supplementary materials). As a result of the obtained data, it can be concluded that the applied GC–MS/MS conditions are acceptable for both the qualitative and quantitative analysis of AAS in plasma samples.

Our recent study [4] shows that the transient matrix effect caused by oleamide can improve the sensitivity of estimating CBD and other xenobiotics in plasma samples. This leads to the question of whether it would be possible to utilise other substances such as polyethylene glycol (PEG-400), tetradecanoic acid (C14-COOH), *n-*tetradecylalcohol (C14-OH) or *n-*tetradecylamine (C14-NH_2_) to induce this phenomenon and, therefore, increase the signal from AAS. The utilisation of transient matrix effect could serve as a way of extending the methods already developed, such as the QuEChERS technique, for the determination of steroids, thus achieving even better analytical results.

To determine which of previously mentioned protectants can cause a matrix effect and, if so, which one would result in the most significant increase in the analytical signal, a series of samples containing AAS solutions no. 1 and no. 2 in ACN containing analytes of a concentration of 4 µg/mL (dromostanolone propionate, methandienone, testosterone for solution no. 1 and dehydroepiandrosterone, testosterone acetate for solution no. 2) each, 40 µg/mL (nandrolone, turinabol for solution no. 1 and fluoxymestrone, trenbolone acetate, trenbolone for solution no. 2) each and 40 µL of protectant also dissolved in ACN (see “Standard solutions” section) were prepared. These solutions were then subjected to the GC–MS/MS analysis, monitoring ions characteristic of the tested analytes. The results of those experiments are shown in Fig. 1. The samples that did not contain a protectant additive were used as the reference points. Owing to the multitude of obtained test results, Fig. 1 includes those obtained for methandienone only.

When employing the matrix effect for analytical purposes, its transient nature is one of the most important aspects. In order to prove both the nature of this phenomenon and a stability of this process, a decision was made to conduct experiment in which a series of samples containing AAS solutions no. 1 and no. 2 in ACN containing analytes of a concentration of 2 µg/mL (dromostanolone propionate, methandienone, testosterone for solution no. 1 and dehydroepiandrosterone, testosterone acetate for solution no. 2) each, 20 µg/mL (nandrolone, turinabol for solution no. 1 and fluoxymestrone, trenbolone acetate, trenbolone for solution no. 2) each and 150 µL of PEG-400 solution in ACN were alternately dosed. Dosing was repeated five times. Owing to the large number of test results obtained, Fig. 2 includes only those obtained for methandienone.

Nevertheless, to guarantee the maximum increase in the analytical signal induced by the addition of the protectant, it is necessary to determine the volume of PEG-400 that represents the optimum value. In order to ascertain this, a series of samples containing AAS solutions no. 1 and no. 2 in ACN containing analytes of a concentration of 2 µg/mL (dromostanolone propionate, methandienone, testosterone for solution no. 1 and dehydroepiandrosterone, testosterone acetate for solution no. 2) each, 20 µg/mL (nandrolone, turinabol for solution no. 1 and fluoxymestrone, trenbolone acetate, trenbolone for solution no. 2) each with the addition of various volumes of protectant solution (in a range of 25 µL to 150 µL). Given the considerable number of test results obtained, Fig. 3 shows only those obtained for methandienone.

The question is therefore: whether the matrix effect can be used to enhance the signal of AAS found in biological material? In order to answer this question, experiments were performed in which a plasma samples were fortified with 100 µL of PEG-400 solution in ACN. The concentrations of substances were 2 µg/mL (dromostanolone propionate, methandienone, testosterone for solution no. 1 and dehydroepiandrosterone, testosterone acetate for solution no. 2) each and 20 µg/mL (nandrolone, turinabol for solution no. 1 and fluoxymestrone, trenbolone acetate, trenbolone for solution no. 2) each. The results for all substances are included in Figs. 4 and 5. Figure 4 shows the detector response values obtained for the examined analytes before and after the addition of the protectant to the plasma samples. The data presented in Fig. 5 show three pairs of chromatograms illustrating the effect of the protectant addition on the analytical signal intensity for two different mixtures of analytes (no. 1 and no. 2). The first pair of chromatograms (A' and A'') represents the profiles obtained for the samples before the protectant addition—for mixture no. 1 (A') and mixture no. 2 (A''). The second pair (B' and B'') shows the chromatograms of the same samples after the protectant addition, again for mixture no. 1 (B') and mixture no. 2 (B''). The third pair of chromatograms (C' and C'') overlays the chromatograms before and after the protectant addition, allowing for direct comparison of the analytical signal intensities and visualizing the enhancement effect. Chromatograms C' and C'' refer to mixtures no. 1 and no. 2, respectively.

The data clearly indicate that the addition of the protectant results in a significant increase in signal intensity for the individual analytes.

The analytical value of the described method was evaluated through a validation procedure. The findings of this process are shown in Table 2 (see “Method validation and statistical analysis” section), which demonstrates that the method exhibits excellent linearity, remarkably low detection limits, and commendable inter- and intraday precision for all of the steroids analysed.

**Table 2:** Linearities, intra- and interday precisions and accuracies, limits of detection (LOD), limits of quantification (LOQ) of examined AAS

Compound	Without protectant (PEG-400) addition						
	Linearity (*R*^2^)	Intraday precision (% RSD)	Interday precision (% RSD)	Intraday accuracy (%)	Interday accuracy (%)	LOD (ng/mL)	LOQ (ng/mL)
Dehydroepiandrosterone	0.9993	3.22	3.69	98.12	97.32	0.28	0.93
Dromostanolone propionate	0.9991	3.31	3.94	97.96	97.11	0.69	2.30
Fluoxymestrone	0.9995	3.16	3.67	98.01	97.37	0.52	1.70
Methandienone	0.9996	3.35	3.82	97.98	97.11	0.36	1.20
Nandrolone	0.9989	4.01	4.51	96.92	95.51	1.21	4.03
Testosterone acetate	0.9990	3.93	4.22	96.55	95.87	1.09	3.63
Testosterone	0.9994	3.29	3.67	97.23	96.78	0.74	2.46
Trenbolone	0.9993	3.74	3.93	98.14	97.10	0.82	2.73
Trenbolone acetate	0.9997	3.01	3.34	98.52	98.01	0.14	0.47
Turinabol	0.9998	3.15	3.55	98.97	98.38	0.16	0.53

With protectant (PEG-400) addition						
Linearity (*R*^2^)	Intraday precision (% RSD)	Interday precision (% RSD)	Intraday accuracy (%)	Interday accuracy (%)	LOD (ng/mL)	LOQ (ng/mL)
0.9995	3.02	3.13	98.97	98.17	0.03	0.10
0.9994	3.11	3.24	98.45	98.01	0.36	1.20
0.9997	2.99	3.09	98.69	98.31	0.10	0.33
0.9997	3.16	3.33	98.47	97.87	0.21	0.70
0.9994	3.41	3.59	97.61	97.12	0.13	0.43
0.9993	3.22	3.47	97.49	97.08	0.49	1.63
0.9996	2,99	3.16	98.55	98.15	0.31	1.03
0.9997	3.55	3.74	98.89	98.36	0.34	1.13
0.9998	2.89	2.99	98.78	98.29	0.05	0.17
0.9999	3.01	3.16	99.07	98.51	0.07	0.23

*R*^2^ coefficient of determination, *RSD* relative standard deviation

## Discussion

The use of performance-enhancing substances by athletes may not only create a risk of unfair competition, but may also endanger their life or health. For this reason, uniform rules have been created regarding the use of various types of substances that can affect the disposition of athletes during certain competitions [15]. However, the mere existence of these regulations does not guarantee strict compliance. The increasing popularity of, and thus easier access to, doping substances such as AAS requires the improvement of the existing detection methods. One of the more intriguing approaches to enhancing the efficacy of determination methods by increasing the signal of the analytes involves the exploitation of a phenomenon that is often regarded as a negative: the matrix effect.

The matrix effect relates to the interference of components present in the sample matrix on the detection and quantification of the analyte. The factors that may be responsible for these effects are numerous, including the chemical composition of the matrix, the physical properties of the sample, and the interactions between the sample components and the chromatographic system. The impact of the matrix effect on GC analysis can be both positive and negative, depending on the nature of the matrix and the analyte of interest. An example of a positive utilisation of this phenomenon is so-called transient matrix effect.

Although the enhancement of the signal from analytes using components present in the sample matrix has been attempted in a variety of ways [16, 17], the transient matrix effect retains its uniqueness due to its temporary nature. The influence of the factor (so-called protectant) that causes it is not continuous. Consequently, when a series of samples containing additives of a protectant is tested, the effect on the signal of the analytes will not overlap. It is possible that the mechanism behind the increase in signal is a chemical interaction between the analyte and the protectant. For instance, high-boiling matrix components that have been evaporated in the liner initially can create a region at the beginning of the chromatographic column where analytes with an affinity for them would concentrate. This, in turn, will result in a concentration of the analytes' concentration zone, which will increase analytical signal and sharpen peaks.

Nonetheless, prior to the utilisation of the transient matrix effect for the purposes mentioned above, the structure of the substances to be determined must be taken into consideration. The chemical structures and fragmentation pathways of AASs (testosterone, nandrolone and methandienone) are illustrated in Fig. 1S. The optimal conditions for the instrumentation used were determined in the laboratory for each compound. The MRM transitions and optimal collision energies of the examined compounds are collected in Figs. 2S, 3S and Table 1.

The selection of a suitable sample preparation technique is one of the most important steps in the entire analytical procedure. The success of the entire analytical process depends on it. QuEChERS is an extraction technique characterised by the usage of inorganic salts and extractant in the extraction step of the procedure and solid sorbent in the sample purification step. The decrease of mutual solubility of ACN and water and the increase of analyte recovery are the main purpose of inorganic salt addition. Hence, the QuEChERS optimisation should include the effects of the NaCl, MgSO_4,_ ACN, sample volume and solid C-18 sorbent amounts on analyte recoveries. The influence of their amounts, used in the QuEChERS procedure on the AAS recoveries in plasma sample is shown in Fig. 4S. The effect of each factor on the recoveries of the analytes was determined when other variables were held constant. As can be seen, each factor had a significant effect on the recoveries of the analytes tested. Furthermore, the effect of each factor varied at different constant levels of the other QuEChERS variables. The optimal conditions for QuEChERS were found to be as follows: NaCl -  87.5 mg, MgSO_4 _ - 350 mg, ACN -  500 µL (the optimal volume of ACN was initially set at 700 µL; however, this was subsequently reduced to 500 µL due to limitations in mixability, which were a consequence of the size of Eppendorf tube), plasma volume -  875 µL and C-18 sorbent -  17.5 mg (see "QuEChERS sample preparation method" section). For more practical application of the method, the end user may apply certain rounding of sample volume and sorbent mass -  for instance, using 900 µL of plasma volume and 20 mg of C-18 sorbent. Once the final QuEChERS procedure has been developed, it is possible to further improve it to obtain the best analytical results. One possible strategy that can be employed for this purpose is to extend it to include the phenomenon of the transient matrix effect.

The implementation of a transient matrix effect, utilizing oleamide as protectant to enhance the GC signal from CBD and other xenobiotics in plasma samples [4], is one example. The strength of the observed effect can be attributed to a combination of factors, including the specific properties of the protectant, the analytes, and the mutual concentration of these substances. If its effectiveness in improving the determination of CBD and other xenobiotics has been confirmed, the question arises as to whether a similar effect can be achieved utilizing other substances as protectants in the case of the analysis of AAS, and, if so, what would be its impact?

The results of experiments in which test samples containing steroids were fortified with different types of substances (polyethylene glycol (PEG-400), tetradecanoic acid (C14-COOH), *n*-tetradecylalcohol (C14-OH) or *n*-tetradecylamine (C14-NH_2_)) in order to determine their capacity to induce a matrix effect and their impact on the analytical signal of methandienone (see Results section) are shown in Fig. 1. The conclusions that can be drawn from the data illustrated there are as follows:

- The addition of each type of protectants results in an increase in the GC–MS signal from the AASs;
- The smallest signal increase, reaching 151% was observed when *n-*tetradecylalcohol was used as a protectant;
- The most significant enhancement in the analytical signal was observed upon the incorporation of PEG-400 as protectant, with an increase of 178% being observed.

In the case of utilizing the matrix effect for analytical purposes, its transient nature is one of the most important aspects. In the transient (one-off) matrix effect, the phenomenon of increasing the analytical signal of the tested compound will not be overlaid when subsequent samples containing a protectant are injected. That means that the results of chromatographic analysis obtained from individual alternately injected samples should be analogous. This property offers significant opportunities for the creation of a controlled transient matrix effect. The findings of the experiment demonstrated that the transient matrix effect was only temporary and that the process was indeed stable. These outcomes are illustrated in Fig. 2. The analysis of the results presented shows that subsequent alternate sample injections show identical detector response within a given sample type. This finding confirms that the matrix effect produced is a transient matrix effect. Furthermore, the protectant is not affecting the subsequent sample injections as it leaves the chromatographic system during the analysis. This is a crucial point for the future applicability of the method.

Once the usefulness of the transient matrix effect has been demonstrated, it is important to consider how its efficacy might be maximised in the case of enhancing the analytical signal from AAS. For the purpose of determining the optimum volume of protectant that causes this phenomenon, an experiment was conducted. The experimental findings (shown in Fig. 3) indicated that a volume of 100 µL represents the optimal value, beyond which adverse effects begin to manifest.

Following the confirmation of the effectiveness of the matrix effect for test solutions, the subsequent step is the demonstration of its applicability to real samples. The most significant aspect from the standpoint of preventing doping in sport is the determination of steroids in biological matrices such as plasma. If the increase in analyte signal that was observed for test solutions also occurred during the determination of steroids extracted from plasma samples, it would be possible to reduce both the LOD and LOQ of steroids within the bodies of athletes.

The experiment was conducted to ascertain the practical value of utilizing the matrix effect when determining steroids in biological matrices, such as plasma samples. To this end, MRM chromatograms of plasma samples fortified with AAS mixtures, which had undergone the standard QuEChERS procedure, were compared with those obtained after the plasma samples had been subjected to the procedure extended by the use of the transient matrix effect. The results of experiments utilizing the properties of PEG-400, the most effective of the proposed protectants, are presented in Figs. 4 and 5. The following conclusions can be drawn from the data illustrated there:

- A similar to the case of test samples, the addition of PEG-400 as protectant to generate transient matrix effect results in an increase in the GC–MS signal from the AASs in biological matrices, such as plasma samples;
- The magnitude of the signal increase is contingent on the type of steroid being assayed;
- For the majority of the steroids that were examined in the study, the signal increase around 200% was observed;
- The highest increase in the analytical signal was observed for nandrolone, the determination of which during the standard QuEChERS procedure may present certain difficulties. However, following the extension by the addition of a protectant, the signal values reach values of 912% of the reference point.

It is crucial to note that the obtained transient matrix effect demonstrates the high usefulness of the implemented method and has a significant impact on the enhancement of the analytical signal of AAS in plasma. The utilisation of this method for the analysis of complex biological samples is a successful extension of the basic QuEChERS technique.

## Conclusions

The term "matrix effect" refers to interference from components in the sample matrix that can impact the detection and quantification of the analyte. This effect can be either positive or negative, depending on the matrix and analyte. This article presents a positive use of the "transient matrix effect" to enhance the analytical signal of AAS in plasma samples, including dehydroepiandrosterone, drostanolone propionate, fluoxymestrone, methandienone, nandrolone, testosterone acetate, trenbolone acetate, testosterone, trenbolone, and turinabol. A procedure was developed to isolate the analytes using the QuEChERS technique, which was further extended by adding protectants (e.g., polyethylene glycol (PEG-400), tetradecanoic acid (C14-COOH), *n-*tetradecyl alcohol (C14-OH), and *n-*tetradecylamine (C14-NH_2_)) to initiate the transient matrix effect.

The results show that PEG-400 provided the highest signal increase for methandienone (178%), followed by C14-NH_2_ (174%), C14-COOH (167%), and C14-OH (151%). PEG-400’s high boiling point and its affinity for the analytes, results in its initial condensation on the chromatographic column, creating a space at its beginning where analytes concentrate. This results in both sharper and enhanced signals.

The extent of the signal increase achieved by the QuEChERS procedure extended with PEG-400 varies depending on the analyte. For dehydroepiandrosterone, the signal increased by 248%, for drostanolone propionate by 190%, for fluoxymestrone by 510%, for methandienone by 175%, for nandrolone by 912%, for testosterone acetate by 221%, for trenbolone acetate by 288%, for testosterone by 288%, for trenbolone by 239%, and for turinabol by 252%. These results demonstrate the varying degrees of interaction between the analytes and PEG-400.

## Supplementary Information

Below is the link to the electronic supplementary material.

Supplemental Figure 1 Chemical structures and fragmentation pathways of (**A)** testosterone, (**B) **nandrolone and (**C)** methandienone.

Supplemental Figure 2 Collision energies (CE) optimization for quantitative MRM transitions of (**A) **dromostanolone propionate, (**B)** methandienone, (**C)** testosterone, (**D)** nandrolone and (**E)** turinabol.

Supplemental Figure 3 Collision energies (CE) optimization for quantitative MRM transitions of (**A) **dehydroepiandrosterone, (**B)** testosterone acetate, (**C)** fluoxymestrone, (**D)** trenboloneacetate and (**E)** trenbolone.

Supplemental Figure 4 The optimisation of the QuEChERS sample preparation procedure. Determining the optimal amounts of individual additives to increase analyte yield – extractant volume (**A)**, the amount of extraction salts (**B)**, plasma sample volume (**C)** and the amount of sorbent (**D)** – using methandienone as an example of examined AAS.

Supplemental Figure 5 The MRM chromatograms of a blank blood plasma samples (A’ and A’’) and blood plasma samples spiked with AAS solutions no. 1 and no. 2 in ACN (B’ and B’’) after QuEChERS sample preparation procedure.

## Declarations

### Data availability

All data generated or analyzed during the study are included in the present article.
