# Supplementary figures and images for: Application of the transient matrix effect for determination of anabolic–androgenic steroids in biological samples by GC–MS/MS
Source: Forensic Toxicol. 2025 Jun 26;44(1):204–16. doi: 10.1007/s11419-025-00731-6 (PMC12858534; doi:10.1007/s11419-025-00731-6)

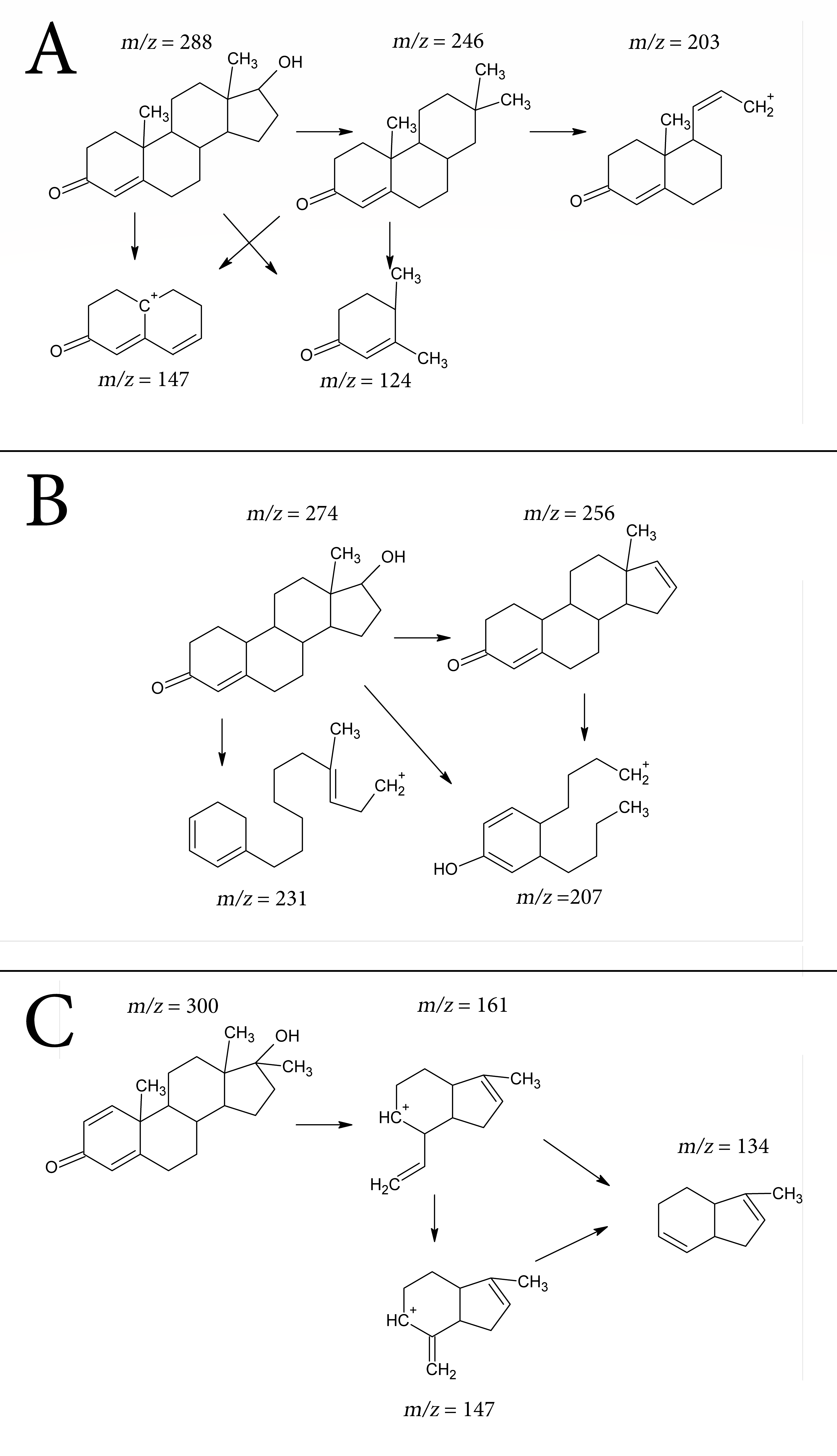

Supplement: Supplementary file 1 — Chemical structures and fragmentation pathways of (A) testosterone, (B) nandrolone and (C) methandienone. [file 11419_2025_731_MOESM1_ESM.tiff]

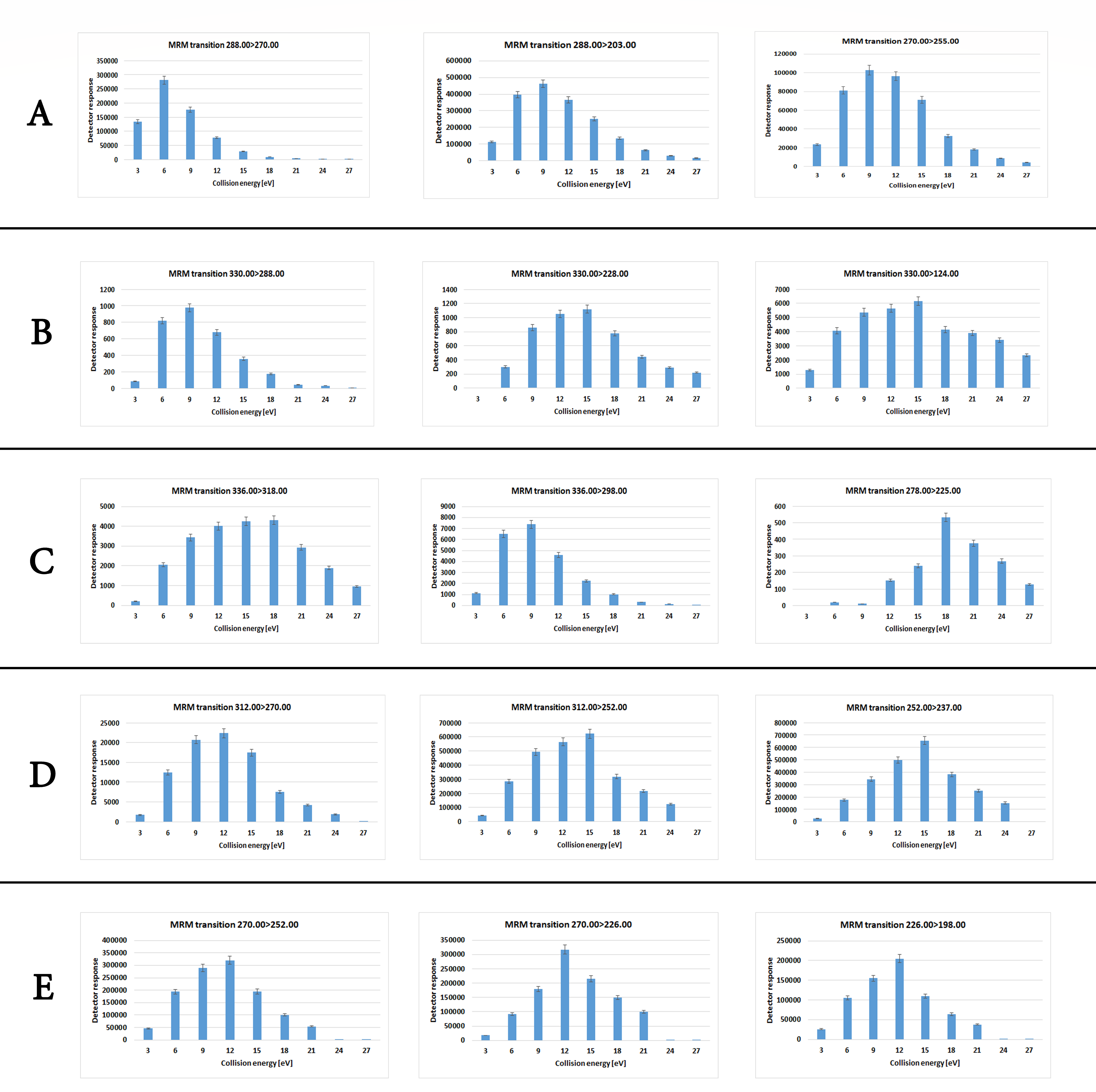

Supplement: Supplementary file 3 — Collision energies (CE) optimization for quantitative MRM transitions of (A) dehydroepiandrosterone, (B) testosterone acetate, (C) fluoxymestrone, (D) trenboloneacetate and (E) trenbolone. [file 11419_2025_731_MOESM3_ESM.tiff]

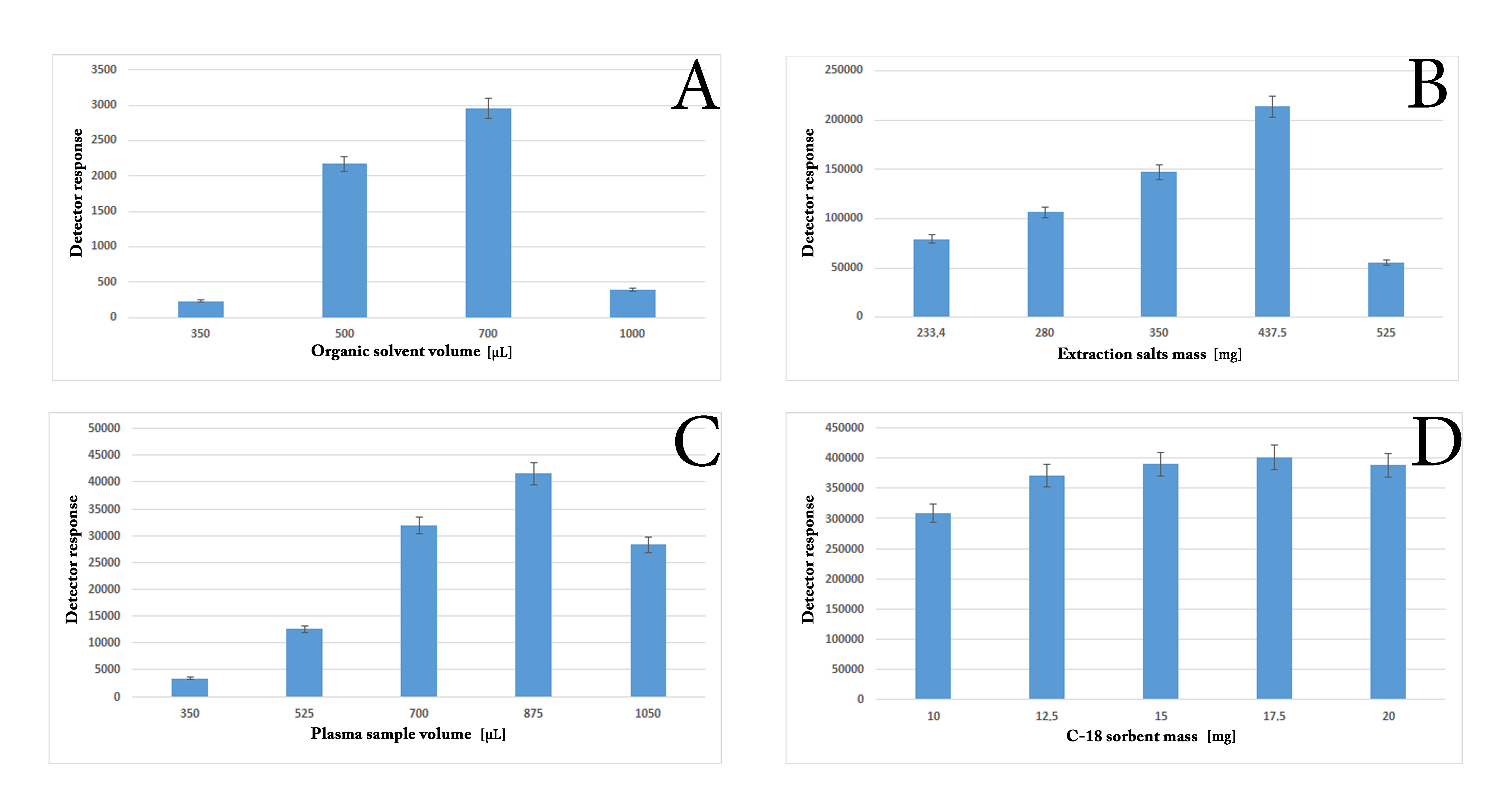

Supplement: Supplementary file 4 — The optimisation of the QuEChERS sample preparation procedure. Determining the optimal amounts of individual additives to increase analyte yield – extractant volume (A), the amount of extraction salts (B), plasma sample volume (C) and the amount of sorbent (D) – using methandienone as an example of examined AAS. [file 11419_2025_731_MOESM4_ESM.tif]

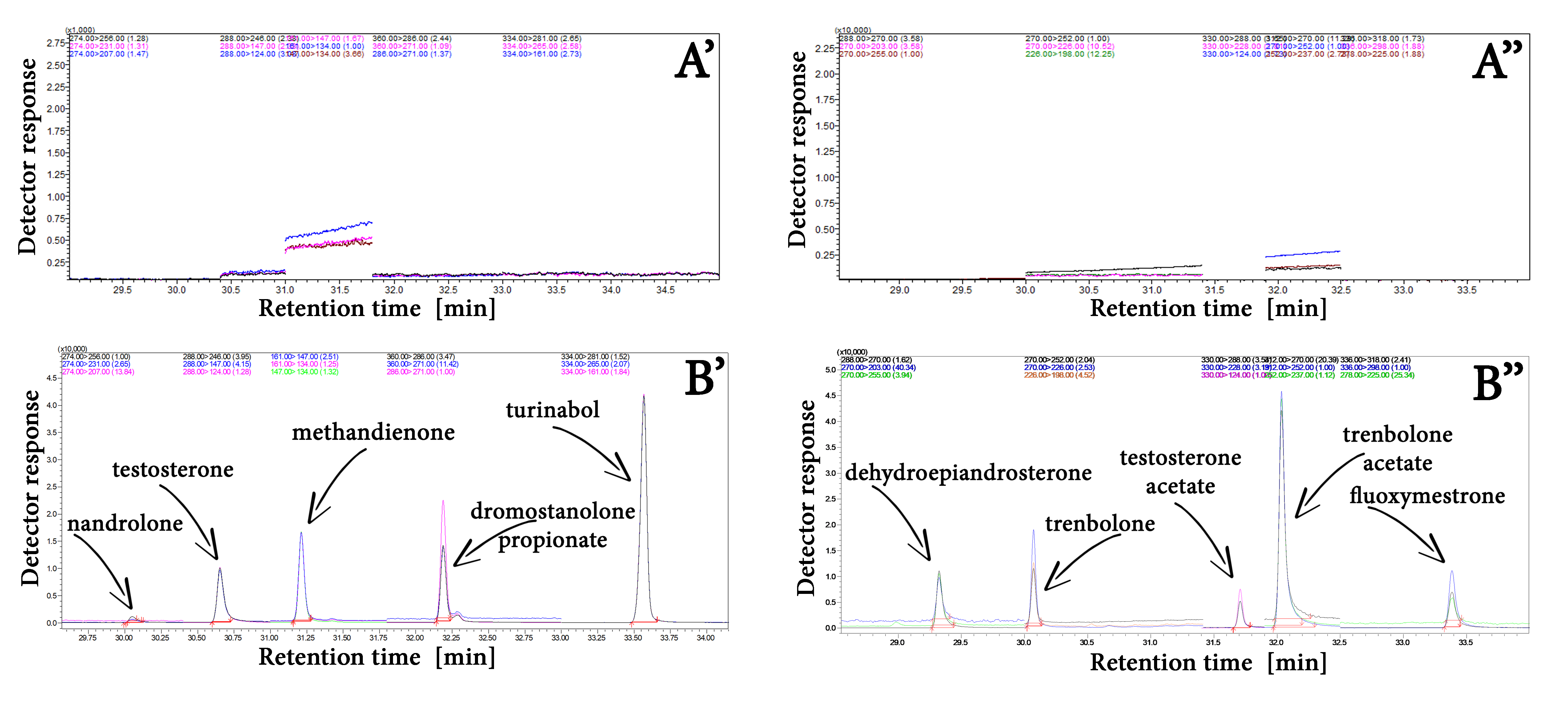

Supplement: Supplementary file 5 — The MRM chromatograms of a blank blood plasma samples (A’ and A’’) and blood plasma samples spiked with AAS solutions no. 1 and no. 2 in ACN (B’ and B’’) after QuEChERS sample preparation procedure. [file 11419_2025_731_MOESM5_ESM.tif]
